# Puerperal Fever; in What Way It May Originate from Erysipelas, and How Communicable

**Published:** 1848-05

**Authors:** R. U. West

**Affiliations:** Surgeon, Hogsthorpe, Lincolnshire


*Puerperal Fever; in what way it may originate from Erysipelas, and how communicable.* By R. U. West, Surgeon, Hogsthorpe, Lincolnshire.—The following cases, which are corroborative of the opinion that puerperal fever may originate from attendance on erysipelatous patients, and that a malady so originating is communicable from one puerperal woman to another, are also suggestive of other conclusions on this painful subject, which are, I think, of some practical importance.

On the 6th of January, 1842, I was called in to attend an elderly gentleman in this village, who had erysipelas of the face. As yet his symptoms were not at all urgent; it appeared to be quite a slight and common case. I sent some saline medicine, and a lotion. The same evening I attended a case of midwifery, two miles from my residence. The next morning, before visiting my erysipelatous patient, I attended a second case of midwifery, four miles from my residence. On my return home, I found my patient considerably worse; I applied nitrate of silver freely all over the affected part. At one or two o'clock in the morning of the next day, January 8th, I was called up to him. He was much worse; face and head frightfully swollen down to the shoulders on both sides. After visiting him again the same morning at nine o'clock, I called upon both my lying-in patients. They were doing well, and in fact recovered without a single bad symptom. In the evening I found the old gentleman in a very bad way; there was some delirium, and the head and face were still more swollen. I made a few punctures in both cheeks, and while busy with him was sent for in all haste to attend a case of midwifery,—Mrs. T. T. of L——, a distance of three miles. I believe I went without washing my hands. I was not detained more than a couple of hours, and as I was riding homewards, I was met by my groom, who had been sent to me with the announcement that an alarming change had taken place with my erysipelatous patient. I hastened on, and found him comatose, with a dusky livid appearance of the face. He died in the course of the night, a frightful spectacle.

And now commenced my troubles. In the night of the 9th of January, or rather early in the morning of the 10th, I was again summoned to Mrs. T. T. I found her in the following state:—Very severe pain, aggravated in paroxysms, like after-pains; some abdominal tenderness; had had a shivering fit; some headache; no anxiety of countenance; pulse 120, soft and full; skin hot; diaphoresis; lochia abundant; no signs of milk, but as it was barely thirty hours after delivery, it was quite early enough for that. I ordered fomentations, and sent some doses of Dover's powder in a saline mixture. Eight hours after, pulse 100; felt much better. Sent her an opening powder,—calomel and rhubarb.

January 11. Pulse 112; more pain again, with tenderness; *complained much of pain and heat of the vulva.* Ordered eight leeches to the abdomen, and four to the vulva, and gave her a grain of calomel and three grains of Dover's powder with saline mixture every four hours.

12th. Tenderness increased; abdomen tympanitic; pulse 130, smaller, but not sharp; some anxiety; lochia still abundant; no milk; tongue white and slimy. I proposed venesection, but finding the husband inclined to be dissatisfied, I made him fetch my friend Dr. Barker, of Spilsby, with whose sanction I bled the patient. The medicines were continued.

13. Pulse below 100; pain better; abdomen very full; did not pass sufficient water.

She continued to improve till January 16th, though very nervous and timid, and on that account giving a good deal of trouble, but in the evening of that day I found it necessary to pass the catheter, which had to be repeated once or twice a day until the 26th, when the retention, after resisting various medicines, such as Tinctura Lyttæ, strychnine, mucilage with Liquor Potassæ, &c., yielded at once to the Tinctura Ferri Muriatis, in doses of fifteen drops every four hours. The pulse never got above 100 after the venesection. She continued weakly for about a month.

On the 15th of January, at one o'clock in the morning, I attended a case of midwifery in a village about six miles from my residence. The woman had "got it over" before my arrival; I had not even occasion to remove the placenta. I, however, felt her pulse, of course, besides ascertaining, by pressing the abdomen, that the uterus was safely contracted, which I never omit to do before leaving a lying-in woman.

On the 17th I called upon this patient again, after having in the morning passed the catheter for Mrs. T. T. She was getting on well, and *recovered without the occurrence of a single unpleasant symptom.*

In the evening of the 16th of January, immediately after my return home from a visit to Mrs. T. T., on which occasion, be it remembered, I had been obliged to use the catheter, I was summoned to attend Mrs. R. B., of C., about a mile from my residence. I was with her about an hour before she was delivered; it was her fourth child, and her labour was easier and quicker than common.

On the 18th, in the morning, being about thirty-six hours after her delivery, I was again summoned to this patient. She was suffering very severely from constant pain in the abdomen, aggravated in paroxysms, (which I have generally found to be the case in *puerperal* peritonitis;) there was very considerable tenderness on pressure; pain in the forehead; pulse 130, small and sharp; respiration hurried; countenance flushed and anxious; restlessness; lochial discharge slight; bowels confined; some nausea; tongue white; skin hot; could not find that she had had any rigors. I bled her in the arm immediately, after which the pulse dropped to 100, and the pain was somewhat abated. Sent an opening powder, and some fever medicine.

When I called in the evening the aperient had not acted; the pain had returned as bad as ever, the tenderness being excessive; pulse 130, as at first. I took some more blood from the arm, and gave her an enema, which brought away some scybala. The pains were now materially relieved, though the pulse did not drop this time. Sent an opening mixture, with Magn. Sulph. and Tinct. Hyoscyami.

19th. Felt better; purged two or three times; no return of paroxysmal pains; tenderness still remaining; pulse 130. The milk had appeared in the breasts, which I looked upon as favourable.

20th. Complete retention of urine, causing pain and tenderness again. Passed the catheter, which gave great relief. In other respects better.

21st. No water passed since the catheter was used at noon the day before, catheterism therefore necessary both morning and evening; there was still *malaise*, though the symptoms were apparently less urgent; there was still pain, with abdominal tenderness; and the pulse was never below 130. I therefore this day gave calomel and opium in pills, and applied a dozen leeches to the abdomen.

The next morning, January 22d, the catheter was not needed; there was some tendency to diarrhœa, and she had a severe rigor. I found all the symptoms aggravated. Any further depletion I could not venture upon; on the contrary, I sent some aromatic confection, continuing the calomel.

I saw her twice or three times on this day. I was called up the next night, and finding the result likely to be fatal, persuaded the husband to fetch Dr. Barker. By the time he arrived, in the course of the morning of the 23d, the poor woman was beginning to be typhoid; she had been at times delirious; there was tympanitic distension of the abdomen, with other untoward symptoms. We gave carbonate of ammonia, to which was added wine the next day.

On the 25th she was quite typhoid; black tongue; subsultus tendinum distressingly frequent; hiccup, &c.; and in the evening of the same day she sank. A constipated state of the bowels in the first instance seemed to complicate the disease unfavourably. There were some scybala voided even on the 23d. The milk continued in the breast to the last; its appearance, therefore, in this disease, is not critical.

Dr. Barker and I discussed the matter most anxiously while he was with me on the 23d, and although we were both of us quite unconscious that the case of erysipelas had had anything to do with the puerperal affection, we felt compelled to the decision that Mrs. R. B.'s case was genuine malignant puerperal fever, and that as it had probably been communicated from Mrs. T. T., my only safe course to prevent its further spread would be to discontinue attending midwifery for a time. I therefore at once sent for a substitute. But before his arrival (I had 23 miles to send,) I was *obliged* to attend another midwifery case. Oh! how anxious I was. I changed all my clothes before going; washed my hands in several waters, with solution of chloride of lime, and although I delivered the woman, I was careful to make as few examinations as possible. This case, it will be understood, was attended on the 23d January. The woman had some slight symptoms of peritonitis on the 25th; her pulse was very frequent, and there was severe pain with some tenderness. She had some saline aperient medicine, with fomentations, and did well.

As soon as I could be released from my attendance on Mrs. T. T., —for I did not think it prudent to allow my substitute to pass the catheter for her,—I went into Leicestershire for a fortnight. I am happy to say that I have not seen a case of malignant puerperal fever since, though I have, as may be supposed, seen several cases of simple peritonitis.

I may be allowed to observe upon the above accurate detail of facts, that if it be conceded that my attendance on the case of erysipelas was the cause of this formidable malady getting into my practice, which, painful as the thought may be, I think was the case, it seems probable—

*First.* That as the cases attended on the 6th and 7th of January, recovered without the appearance of any unfavourable symptoms, a form of erysipelas, which may subsequently in its *malignant* and almost *putrid* stage originate puerperal fever, will not do so in its *early* stage.

*Secondly.* That as the disease did not come on in the same patients, notwithstanding my visiting them after attending the erysipelatous case when the symptoms *had* become formidable, the merely taking a lying-in woman by the hand will not originate the disease.

*Thirdly.* That as the woman attended on the 15th of January recovered without any symptoms of the malady, although I might at the time, be supposed to be tainted with both the erysipelas and the puerperal disease of Mrs. T. T., it requires something more than a mere *visit* to a parturient woman to communicate the disease from one puerperal patient to another.

*Fourthly.* That, bearing in mind the above suppositions, the circumstances immediately preceding and accompanying the attendance on both Mrs. T. T. and Mrs. R. B., go to prove that it requires actual contact of the accoucheur's infected hand with the mucous membrane of the vagina, both to originate and communicate the disease. (Query—would not the heat and pain of the vulva in the first case confirm or strengthen this supposition?)

*Fifthly.* That the slight manner in which the disease appeared in the woman attended on the 23d, (notwithstanding I was still passing the catheter daily for Mrs. T. T., and had but just returned from a visit to Mrs. R. B., the latter being at the time almost typhoid,) would serve to prove that ablution, change of clothes, chlorides, &c., are of some little avail.

*Sixthly.* That erysipelas may originate a *mild* form of puerperal fever, which may in its turn communicate a *more malignant* form. (In the two cases here alluded to, the symptoms in the commencement were nearly the same, the occurrence of dysuria in them both being somewhat remarkable; the fatal difference was in their *terminations*, —typhus and death in the one, gradual recovery in the other.)

And, *lastly*, that a puerperal fever, originating from a case of erysipelas, is not *necessarily fatal.*

I offer the above remarks with considerable diffidence. The *facts* I have detailed may be relied on, and I leave the reader to found upon them what conclusions he may please.—*Prov. Med. and Surg. Jour.*

